# Levetiracetam Therapeutic Drug Monitoring in a Large Cohort of Korean Epileptic Patients

**DOI:** 10.3390/ph14080826

**Published:** 2021-08-23

**Authors:** Changhee Ha, Hyun-Seung Lee, Eun Yeon Joo, Young-Min Shon, Seung Bong Hong, Dae-Won Seo, Soo-Youn Lee

**Affiliations:** 1 Samsung Medical Center, Department of Laboratory Medicine and Genetics, Sungkyunkwan University School of Medicine, 81 Irwon-ro, Gangnam-gu, Seoul 06351, Korea; chha8808@naver.com (C.H.); ctmasaru@gmail.com (H.-S.L.); 2 Neuroscience Center, Samsung Medical Center, Department of Neurology, Sungkyunkwan University School of Medicine, 81 Irwon-ro, Gangnam-gu, Seoul 06351, Korea; ejoo@skku.edu (E.Y.J.); youngmin.shon@samsung.com (Y.-M.S.); sbhong@skku.edu (S.B.H.); 3 Samsung Medical Center, Department of Clinical Pharmacology and Therapeutics, Sungkyunkwan University School of Medicine, 81 Irwon-ro, Gangnam-gu, Seoul 06351, Korea

**Keywords:** therapeutic drug monitoring, drug–drug interaction, antiepileptic drugs, epilepsy, levetiracetam, pharmacokinetics, Korea

## Abstract

Levetiracetam is a new antiepileptic drug (AED) used for treating and preventing partial or generalized seizures. The usefulness of levetiracetam therapeutic drug monitoring (TDM) is related to inter- or intra-individual pharmacokinetic variability, drug interactions, and patient noncompliance. We aimed to investigate the levetiracetam TDM status in Korean epilepsy patients. Serum trough levetiracetam concentrations were measured using liquid chromatography–tandem mass spectrometry in 710 samples from 550 patients. The median (range) daily and weight-adjusted levetiracetam doses were 1500 (20–5000) mg and 25.5 (3.03–133.0) mg/kg, respectively. Patients on levetiracetam monotherapy constituted only 19.5% of the population, while 30.1% were on co-medication with valproate and 56.0% with enzyme-inducing AEDs (EIAEDs). Observed levetiracetam concentrations were widely distributed, ranging 0.8–95 mg/L, with a median of 17.3 mg/L. Levetiracetam concentrations were therapeutic, supra-therapeutic, and sub-therapeutic in 58.5% (*n* = 393), 11.6% (*n* = 78), and 29.9% (*n* = 201) of samples, respectively. There was a strong correlation between weight-adjusted levetiracetam dosage and concentrations (*ρ* = 0.6896, *p* < 0.0001). In this large-scale clinical study, a large inter-individual difference in levetiracetam pharmacokinetics was observed, and levetiracetam concentrations were influenced by EIAEDs. For individual dose adjustments and monitoring compliance, routine levetiracetam TDM is needed in epilepsy patients.

## 1. Introduction

Levetiracetam (LEV) is a second-generation antiepileptic drug (AED) approved for use in the treatment of refractory partial seizures with or without secondary generalized seizures in an oral formulation (age ≥ 1 month) and for intravenous use (age ≥ 16) [1].

LEV is absorbed rapidly, reaching peak concentrations within 1 h after intake with almost complete oral bioavailability [2]. Plasma protein binding degree is <10% [2,3]. LEV exhibits first-order kinetics following a single dose and during long-term administration with excretion primarily through kidneys [4]. Half-life of LEV is 6–8 h in healthy adults, reaching steady states within 48 h [4,5,6,7]. Metabolism of LEV is not dependent on the hepatic cytochrome P450 system, whereas the acetamide group of plasma hydroxylase is involved [2,6]. In addition, LEV has a low side-effect rate, a wide daily dose range (250–5000 mg), and favorable patient compliance [3,4], being a valuable treatment option for acute seizure, critically ill patients [8,9,10], and patients in the outpatient department.

Since LEV has favorable pharmacokinetic properties, it is commonly used as an adjunctive therapy for epilepsy [4,11]. Some studies have shown low pharmacological drug interactions with other AEDs, including carbamazepine, phenobarbital, valproate, gabapentin, and lamotrigine [2,11,12,13]. In contrast, other studies have shown AED inducers, including phenytoin, carbamazepine, and oxcarbazepine, increased LEV clearance and affected serum LEV concentrations [4,14,15,16]. Consistent findings, along with a shorter half-life, were observed in patients receiving AED inducers compared to patients on AED non-inducers [11]. LEV clearance is also influenced by age and renal function. In children with epilepsy, the half-life of LEV was shorter and clearance rates were higher than those in adults [17]. On the contrary, the half-life was longer and clearance rates were lower in elderly patients, compared to those in young adults and children [5,18]. In patients with renal impairment, LEV clearance was decreased in all ages [5,16,18,19]. 

Due to the pharmacokinetic characteristics of LEV, including a wide reference range and low side-effect rate, therapeutic drug monitoring (TDM) of second-generation AEDs is not performed routinely in clinical settings [19]. However, considering the inter-patient variability of (both older and newer) AEDs [20], TDM could serve as an individualized therapy especially in patients on AED poly-therapy, geriatric patients, and those with underlying renal diseases. In the clinical management of AED, TDM is useful due to the significant inter- or intra-individual pharmacokinetic variability of drugs caused by underlying diseases (kidney or liver), pharmacogenetic factors, or drug–drug interactions [7,21,22]. Other benefits of TDM are related to drug compliance, in cases where patients skip medication during the absence of epileptic events [7,22]. However, relevant studies of LEV TDM have not been reported within the Korean population. In addition, large-scale studies are meaningful toward furthering patient management. In this study, we investigated the current status of LEV TDM as well as the influence of other AEDs in Korean patients with epilepsy.

## 2. Results

### 2.1. Demographic Characteristics and Descriptive Data

**Figure 1:**
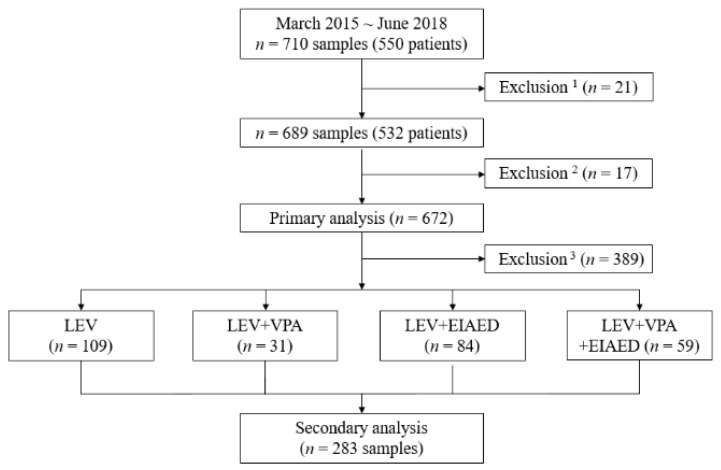
Study workflow. Abbreviations: LEV, levetiracetam; VPA, valproic acid; EIAED, enzyme-inducing antiepileptic drug. ^1^ Incomplete or unavailable medical record. ^2^ Below the lower limit of quantification. ^3^ Pediatric/elderly patient samples, intravenous injections, non-steady state samples, follow-up loss, low compliance, dose change, underlying liver and/or kidney diseases in addition to epilepsy.

A total of 710 samples (from 550 patients) was measured for serum LEV concentrations. Among them, 21 samples (from 18 patients) were excluded due to incomplete data, as were 17 samples (from 13 patients) with LEV concentrations below the lower limit of quantification (LLOQ). As a result, 672 samples from 519 patients were included in the analysis of this study. The workflow of the current study is illustrated in Figure 1.

The median (range) age was 35 (2–88) years. LEV concentration was primarily requested from the outpatient neurology clinic (77.8%), and most patients were administered LEV in tablet form (94.6%). Serum LEV concentration was obtained at a steady state among 98.8% of total samples. LEV concentration was measured only once in the majority of patients (81.7%), with a maximum of 21 times in a single patient. Of note, this patient showed a four-fold variation in LEV concentration, even though the daily LEV dose remained unchanged. More detailed demographics and descriptive data are shown in Table 1.

### 2.2. Levetiracetam Dosing and Serum Levetiracetam Concentrations

The median (range) and weight-adjusted daily LEV doses were 1500 (20–5000) mg and 25.5 (3.03–133.0) mg/kg, respectively. Observed LEV concentrations were widely distributed, ranging from 0.8 to 95 mg/L with a median of 17.3 mg/L. Serum LEV concentrations were therapeutic, supra-therapeutic, and sub-therapeutic in 58.5% (*n* = 393), 11.6% (*n* = 78), and 29.9% (*n* = 201) of samples, respectively. Patients on LEV monotherapy comprised only 19.5% (*n* = 101) of the total, whereas a majority of patients were on co-medication with other AEDs, including valproic acid (VPA) and enzyme-inducing AEDs (EIAEDs). More detailed LEV dosing, concentration, and co-medication data are described in Table 2 and Table S1.

### 2.3. Comparison of Concentration-to-Dose Ratio between Concomitant Medication Groups

**Figure 2:**
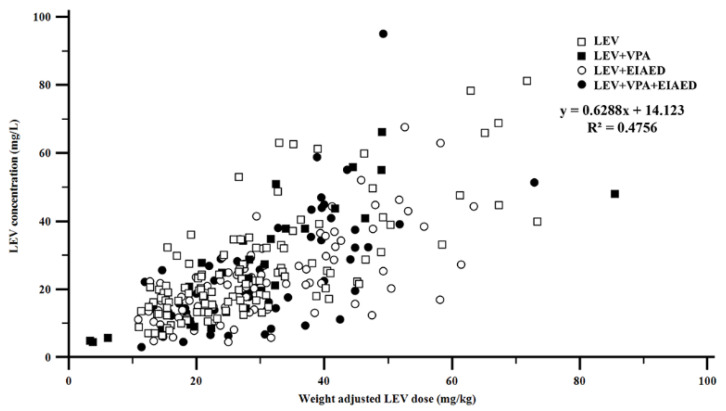
Correlation between weight-adjusted dose and concentration of levetiracetam (*ρ* = 0.6896, *p* < 0.0001, *n* = 283). Abbreviations: LEV, levetiracetam; VPA, valproic acid; EIAED, enzyme-inducing antiepileptic drug.

A total of 283 samples was assigned to four groups (see Methods section). The numbers of samples assigned to each group were 109 (LEV group), 31 (LEV + VPA), 84 (LEV + EIAED), and 59 (LEV + VPA + EIAED), respectively. The median (range) concentration-to-dose ratio (CDR) was 0.81 (0.60–1.08) kg/L. There was a strong correlation between the body weight-adjusted LEV dose and concentrations (*ρ* = 0.6896, *p* < 0.0001) (Figure 2). 

**Figure 3:**
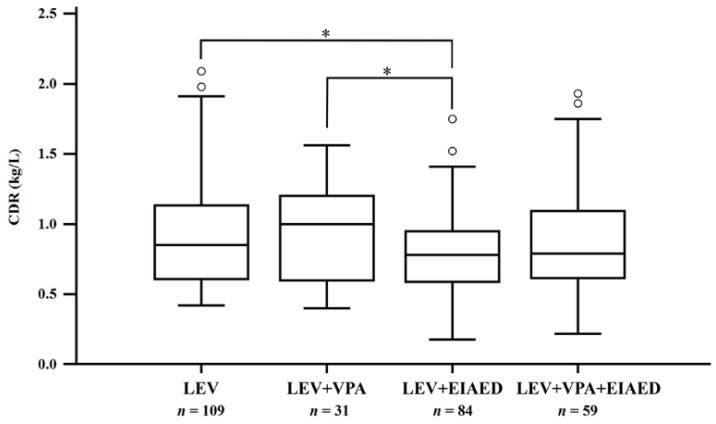
Influence of co-medication on levetiracetam concentration-to-dose ratio (CDR). Group 1, levetiracetam without enzyme-inducing antiepileptic drugs (EIAEDs) nor valproic acid (VPA) (LEV); Group 2, levetiracetam in combination with VPA (LEV + VPA); Group 3, levetiracetam in combination with EIAEDs (LEV + EIAED); Group 4, levetiracetam in combination with both VPA and EIAEDs (LEV + VPA + EIAED). (Conover post-hoc test: * *p* < 0.05.)

Serum LEV concentration and weight-adjusted LEV dose did not reveal a difference among the four groups (*p* > 0.05). However, samples assigned to the LEV group had a reduced LEV dose (*p* = 0.02) compared to those of poly-therapy (LEV + EIAED and LEV + VPA + EIAED groups). CDR was highest in the LEV + VPA group, and lowest in the LEV + EIAED group, with a statistically significant difference (*p* = 0.04) (Figure 3 and Table 3). Gender difference was observed in LEV concentrations (*p* = 0.03) and the weight-adjusted dose (*p* < 0.004, both Mann–Whitney test) but not observed in CDR, or co-medication effect on LEV CDR.

## 3. Discussion

Our study is the largest scale single-center clinical study on LEV routine TDM in epilepsy patients. Even though our data showed a relatively wide dose and concentration range with a high poly-AED therapy ratio, the proportion within the therapeutic range was comparable to other studies, implying the importance of LEV TDM in our center’s clinical settings (Table 4). With the largest dataset, our study showed concomitant EIAEDs affect serum LEV levels.

The significance of the TDM of AEDs has been an essential issue in the field of epileptology for several decades [31]. Concerning pharmacokinetic variability (absorption, distribution, metabolism, and excretion), newer AEDs possess less noticeable inter-individual variability than first-generation AEDs [20,32]. However, according to a pharmacokinetic variability study of four newer AEDs (including LEV), CDR showed a 10-fold inter-individual variability [21]. Our study results also showed extensive inter-individual variability, including daily and weight-adjusted LEV doses, and LEV concentrations with a broader CDR range. Both studies included pediatric, adult, and elderly patients; however, relatively more patients in our study were on AED poly-therapy (80.5%, Table 2) compared to the previous study (50.0%) [21]. Following the common use of LEV as an add-on therapy for epilepsy patients [4,11] and complex interactions between AEDs [33], a higher proportion of poly-therapies would contribute to the wide variability of our data.

According to the therapeutic (or reference) range in our laboratory, almost 30% of samples showed sub-therapeutic levels (Table 1), where 17 samples below LLOQ were excluded before calculation. In interpreting this proportion, which could be meaningful to some degree, a careful evaluation was considered. Quoting the definition of the practice guideline for TDM of AEDs, the lower limit of a reference range is a lower point in which therapeutic effect is likely [7]. Considering the variable drug response and co-medication-LEV drug interactions, although serum LEV levels were below the reference range, the patient could have shown a therapeutic effect. In addition, dose modification was infrequent which almost all samples were collected at a steady state (98.8%, Table 1), supporting the possibility of a therapeutic effect despite low LEV levels. Another study suggested poly-therapy could explain the impaired efficacy of LEV [29]; however, antiepileptic effects could be expressed by other co-medications and only LEV concentrations showed sub-therapeutic levels. Previous studies have shown the efficacy and effect of LEV [13,34,35,36]. However, the relationship between the serum LEV concentration and efficacy is limited, with one study showing LEV to be well tolerated with a low side-effect rate. However, side-effects increase at levels >50 mg/L [37]. Future studies on the clinical manifestations of the epilepsy patients according to LEV concentrations are necessary.

In our study, we excluded other factors (e.g., age, underlying medical conditions) to analyze the pure effect of EIAED and/or VPA on CDR. As a result, the proportion of samples used in the secondary analysis was relatively small compared to the primary analysis (Figure 1). Our single-center study was conducted in a tertiary referral hospital; patients would have underlying diseases in addition to epilepsy, resulting in exclusion in the secondary analysis. Nonetheless, the subgroup analysis showed that co-medication with EIAED lowered CDR compared to both the LEV and LEV + VPA groups (Table 3). Our study results were consistent with the previous studies in which EIAEDs affected the clearance and half-life of LEV [4,15,16]. However, even though the LEV + VPA group had a higher median CDR compared to the LEV group (1.00 vs. 0.85, Table 3), the difference was not statistically significant. The effects of VPA on LEV were inconsistent among previous studies, where VPA derived a modest difference [11] or did not significantly affect LEV concentrations [4,14,29]. The number of samples in the LEV + VPA group was the smallest in our study and did not guarantee a sufficient study power. Another limitation in our study was during sub-group categorization, we only considered whether patients were using VPA and/or EIAED, but not their dose while LEV dose was considered. The variability of co-medications could affect LEV CDR, limiting the statistical analysis.

The consensus guideline 2017 categorized LEV as level four, where LEV TDM potentially is useful [38]. However, specific indications of TDM in this guideline include uncertain adherence, combination treatment, drug–drug interaction of AEDs, and relevant comorbidities [38]. LEV is used commonly as an add-on AED [4,11], and complex interactions between AEDs (including LEV) have been noted [33]. The TDM of AEDs is useful, given its inter- or intra-individual pharmacokinetic variability and drug interactions [7,21,22]. Our results showed a wide inter-individual variation of LEV concentrations and a low LEV monotherapy ratio (Table 2). A majority of the samples was collected from an outpatient clinic (Table 1). A total of 17 samples was excluded due to concentrations below LLOQ and 201 samples (29.9%) showed sub-therapeutic LEV concentrations, which would be due to poor compliance. Concomitant drugs and their dosages might have changed during the long-term AED therapy. To prevent seizures and monitor medication adherence in the outpatient setting, LEV TDM would be indicated. Our patients’ dataset and clinical settings supported the benefits of LEV TDM.

Compared to first-generation AEDs, LEV TDM is limited in study, with a lack of large-scale clinical studies [19]. Previous studies of LEV TDM were based on European populations, with only three studies in Asian populations and no studies in a Korean population [16,23,24]. Additionally, only one large-scale study (> 500 patients) has been published [11]. The accumulation of clinical TDM data from diverse studies of various ethnicities/countries is needed, and we believe that our study is meaningful as such a large-scale study in a new population and a different clinical environment. Through this study, we investigated the dose, concentrations, and co-medication status of Korean patients under LEV treatment and provided large-scale data about the routine use of LEV TDM. A comparison with other large-scale studies is provided in Table 4. However, this study had some limitations. Since this was a retrospective medical review analysis and most patients underwent serum LEV measurement only once, patient information, including efficacy of LEV, dose adjustment, and/or AED change after LEV TDM, was not fully obtained. Considering the lack of studies investigating the relationship between the serum LEV concentration and efficacy, future studies should focus on the clinical manifestations of epilepsy patients with LEV TDM.

## 4. Materials and Methods

### 4.1. Study Subjects

From March 2015 to June 2018, a total of 710 serum LEV concentrations were measured from 550 patients at Samsung Medical Center (Seoul, Korea). Trough concentrations (i.e., drug levels prior to the administration of the next dose) were measured. Dosing history was reviewed to determine steady state. We retrospectively reviewed the patients’ medical records, including route of LEV administration, age, sex, body weight, co-medications (neurologic and/or psychiatric drugs, including other AEDs), and underlying diseases. Patients with loss to follow-up during LEV treatment, incomplete data documentation, and unavailable medical records were excluded (Figure 1). The study protocol was reviewed and approved by the Institutional Review Board of Samsung Medical Center in Seoul, Korea (approval number: 2020-03-099). This research was carried out in accordance with the World Medical Association Declaration of Helsinki. A waiver of informed consent was obtained as the study was a retrospective analysis.

### 4.2. Determination of Levetiracetam Concentration

Serum LEV concentration was measured via high-performance liquid chromatography with tandem mass spectrometry (HPLC-MS/MS). Analyses were performed on an Agilent 6460 tandem mass spectrometer equipped with an Agilent 1260 HPLC system (Agilent Technologies, Santa Clara, CA, USA). HPLC separation was performed on an Agilent Poroshell 120 EC-C18 (3.0 mm × 50 mm, 2.7 µm) using distilled water containing 0.1% formic acid and 2mM ammonium acetate (mobile A) and acetonitrile containing 0.1% formic acid (mobile B) as a mobile phase for gradient elution. The 10 μL supernatant of the obtained simple protein-precipitated sample was injected onto the LC-MS/MS with a flow rate of 0.5 mL/min. Quantitative analysis was performed in multiple reaction monitoring mode (m/z, 177.11 > 154.0 for LEV, 177.1 > 160.1 for internal standard). The linear assay range was 0.5–80 mg/L (R^2^ > 0.99) with an LLOQ of 0.5 mg/L. Intra-day and inter-day coefficients of variation were lower than 10%. The reference LEV range was defined as trough concentrations 10–40 mg/L, and the toxic level was defined as > 100 mg/L [38]. We participated in proficiency testing provided by the LGC Standards Proficiency Testing (LGC Standards Ltd., Bury, UK) in Therapeutic Drugs Scheme.

### 4.3. Data Analysis

For additional sub-group analysis, pediatric (age < 18) or elderly (age > 65) patients, samples from intravenous LEV injections, samples obtained during non-steady state, patients with low compliance, pregnant patients, and those with liver and/or kidney diseases (in addition to epilepsy) were excluded.

To evaluate the effect of concomitant AEDs, patients were separated into four groups of (1) LEV with neither VPA (broad-spectrum inhibitor of drug-metabolizing enzymes) [14] nor EIAEDs such as carbamazepine, oxcarbazepine, phenobarbital, and phenytoin (LEV group); (2) LEV in combination with VPA but without EIAEDs (LEV + VPA group), (3) LEV in combination with EIAEDs but without VPA (LEV + EIAED group), and (4) LEV in combination with both VPA and EIAEDs (LEV + VPA + EIAED group) (Figure 1). Other concomitant AEDs (non-EIAEDs) were not considered for categorization.

Dose-adjusted serum LEV concentrations (CDR) were compared between the four groups. CDR was calculated as follows:

CDR (kg/L) = [LEV concentration (mg/L) / LEV dose per weight (mg/kg)].(1)

To evaluate the CDR difference between the four groups, Kruskal–Wallis test was carried out and Conover post-hoc analysis was performed for differences between specific groups. Statistical analyses were performed using MedCalc Statistical Software version 19.0.5 (MedCalc Software, Ostend, Belgium). Statistical significance was set at *p* < 0.05.

## 5. Conclusions

This was the first large-scale clinical study on LEV TDM in Korean patients with epilepsy. A large inter-individual difference in LEV pharmacokinetics was observed, with a significant proportion of patients having serum LEV levels outside the target reference range and on poly-therapy with other AEDs. These results were related to the pharmacokinetic characteristics of LEV and the use of this drug as an adjunctive therapy. Concomitant EIAEDs lowered LEV CDRs; a careful TDM and dose adjustment were suggested in patients with these co-medications. The results of our study results suggested that a comprehensive understanding of the pharmacokinetic characteristics of LEV and drug–drug interactions of LEV-other AEDs is needed. In addition, routine TDM is required for the individual dose adjustments, monitoring medication efficacy and compliance in order to achieve a personalized treatment for patients on LEV.

## Figures and Tables

**Table 1:** Demographic and descriptive data.

Demographic Characteristics	519 Patients 672 Samples
Patient Data	*n* = 519
No. patients, male:female (%)	275:244 (53.0:47.0)
Age, median (years)	35
Age range (Q25%–Q75%)	2–88 (24–50)
Body weight, median (kg)	63.0
Body weight range (Q25%–Q75%)	1.1–112.5 (53.3–73.0)
Number of measurements in single patient, *n* (%)	
Once	424 (81.7)
Twice	75 (14.5)
Three times or more	20 (3.9)
Mean (range)	1.3 (1–21)
Route of levetiracetam administration, *n* (%)	
PO	491 (94.6)
IV	26 (5.0)
PO and IV	2 (0.4)
Requested department, *n* (%)	
Outpatient department	404 (77.8)
Emergency department	23 (4.4)
Inpatient	92 (17.7)
Sample data	*n* = 672
Sampling period (days) ^1^, *n* (%)	
<2	8 (1.2)
2–7 (one week)	20 (3.0)
8–30 (one month)	45 (6.7)
31–365 (one year)	115 (17.1)
>365	484 (72.0)

^1^ Period between sampling time and initial time of current dose. Abbreviations: Q25%, 25th percentile; Q75%, 75th percentile; PO, oral; IV, intravenous.

**Table 2:** Levetiracetam concentration and medication data.

Characteristics	519 Patients 672 Samples
Sample Data	*n* = 672
LEV dose, median (mg/day)	1500
LEV dose, range (Q25%–Q75%) (mg/day)	20–5000 (1000–2000)
LEV dose per body weight, median (mg/kg/day)	25.5
Range of LEV dose per body weight (Q25%–Q75%)	3.03–133.0 (14.9–38.8)
LEV serum concentration, median (mg/L)	17.3
Range of LEV serum concentration (Q25%-Q75%)	0.8–95.0 (8.5–28.3)
Sub-therapeutic level (<10 mg/L), *n* (%)	201 (29.9)
Therapeutic level (10–40 mg/L), *n* (%)	393 (58.5)
Supra-therapeutic level (>40 mg/L), *n* (%)	78 (11.6)
Patient data	*n* = 519
Number of co-prescribed drugs ^1^, n (%)	
Mean (median)	2.08 (2)
Range (Q25%–Q75%)	0–8 (1–3)
None	101 (19.5)
One	137 (26.4)
Two	99 (19.1)
Three	77 (14.8)
Four	49 (9.4)
Five or more	56 (10.8)

^1^ Neurologic/psychiatric drugs, including antiepileptic drugs. Abbreviations: LEV, levetiracetam; Q25%, 25th percentile; Q75%, 75th percentile; PO, oral; IV, intravenous.

**Table 3:** Serum levetiracetam concentration, dose, and concentration-to-dose ratio for concomitant medication groups (*n* = 283).

Group	Total	LEV (1) ^1^	LEV + VPA (2)	LEV + EIAED (3)	LEV + VPA + EIAED (4)	*p* *	Paired Comparison **
*n*	283	109	31	84	59		
LEV concentration (mg/L) ^2^	21.6 (14.6–32.1)	21.0 (15.6–32.1)	23.1 (11.3–37.8)	21.7 (14.4–28.0)	22.2 (14.4–33.9)	0.9335	
LEV dose (mg/day) ^2^	2000 (1250–2000)	1500 (1000–2000)	2000 (1000–3000)	2000 (1500–2500)	2000 (1425–3000)	0.0217	(1)-(3), (1)-(4)
LEV dose/kg (mg/kg/day) ^2^	27.5 (19.9–38.4)	26.7 (18.4–34.2)	27.8 (19.0–33.6)	28.4 (21.5–40.3)	28.5 (21.4–39.5)	0.2911	
LEV CDR (kg/L) ^2^	0.81 (0.60–1.08)	0.85 (0.61–1.13)	1.00 (0.60–1.20)	0.78 (0.59–0.95)	0.79 (0.62–1.09)	0.0406	(1)-(3), (2)-(3)

^1^ Levetiracetam with non-EIAEDs and psychiatric drugs. ^2^ Values are expressed in median (25th percentile–75th percentile). * *p* values from Kruskal–Wallis test. ** *p* < 0.05 in Conover post-hoc test. Abbreviations: LEV, levetiracetam; VPA, valproic acid; EIAED, enzyme-inducing antiepileptic drug; CDR, concentration-to-drug ratio.

**Table 4:** Previous clinical studies of levetiracetam therapeutic drug monitoring.

Country	Patient (Sample) ^1^, *n*	Age, Years Median (Range) ^1^	Dose, mg/Day Median (Range) ^1^	LEV Conc, mg/L Median (Range) ^1^	Proportion within TR	Proportion of Poly-AED	Concurrent Drugs ^2^	Reference	Year
Korea	519 (672)	35 (2–88)	1500 (20–5000)	17.3 (0.8–95.0)	58.5%	80.5%	CBZ, PB, PHT, OXC	Our study	2021
India	69	6 (1–16)	800 (100–2000)	14.7 (<1–53.8)	NR	60.9%	CBZ, PB, PHT, OXC	[23]	2012
India	330 (348)	NR (0.3–64)	NR	NR (2.4–44.9)	56.9%	63.5%	CBZ, PB, PHT, OXC	[16]	2015
Japan	225 (583)	38 (1–89)	1200 (62.5–3000)	12.5 (0.24–48.8)	NR	90.2%	NS (CBZ, PHT, PB)	[24]	2016
Belgium	94	Mean 10.3 (4–16)	NR	NR	NR	None	NS (CBZ, VPA, TPM, LTG)	[25]	2007
Belgium	228 (Pooled analysis)	9.8 (3 months-18)	NR (20–60 mg/kg/day)	NR	NR	None	CBZ, PHT, PB, PRM	[26]	2008
Germany	297 (363)	33 (2–76)	2500 (250–7000)	14.2 (1.5–48.2)	NR	94.9%	CBZ, OXC, PHT	[4]	2003
Italy	590 (Pooled analysis)	37 (14–70)	2000 (1000–4000)	NR	NR	NR	NS (CBZ, PB, PHT, PRM)	[11]	2003
Italy ^3^	100	NR	2000 (1500–3000) ^4^ 2000 (1312–2500) ^4^	10.4 (7.5–14.0) ^4^ 14.7 (10.7–22.1) ^4^	NR	92.0%	CBZ, PB, PHT	[14]	2004
Italy ^3^	272	NR (30–96)	1750 (1000–2125) ^4^ 1500 (1000–2000) ^4^ 1000 (1000–1500) ^4^	12.7 (8.7–17.2) ^4^ 15.1 (10.0–25.9) ^4^ 23.0 (15.5–29.5) ^4^	NR	100%	CBZ, PB, PHT, OXC	[18]	2012
Spain ^3^	205 (330)	Mean 50.0 Mean 47.9 Mean 41.7	Mean 1892 Mean 2560 Mean 2216	Mean 20.1 Mean 17.3 Mean 20.5	NR	45.5%	CBZ, OXC, PB	[27]	2018
Sweden	103	Mean 10.2 (0–18)	NR	NR	NR	90.3%	CBZ, ETX, PB, PHT, OXC	[28]	2010
Norway	289	Mean 34 (2–93)	NR	NR	NR	NR	CBZ, PB, PHT	[21]	2012
The USA ^3^	308	Mean 25 (16–30) Mean 64 (55–88)	Mean 1990 (250–4625) Mean 1235 (125–4250)	Mean 16.2 (2.5–53.0) Mean 20.0 (3.5–85.3)	NR	89.4%	CBZ, PB, PHT, PRM	[15]	2007
Australia	52	Mean 42 (19–69)	Mean 2919 (250–6000)	Mean 28 (2–100)	61.5%	55.8%	CBZ	[29]	2014
Australia	130	71	1500 (250–4000)	16.2 (9.8–26.1) ^4^	50.8%	NR	NR	[30]	2020

^1^ Described if reported. ^2^ Antiepileptic drugs affecting levetiracetam concentration. ^3^ Study’s subgroup results. ^4^ 25th percentile–75th percentile. Abbreviations: LEV, levetiracetam; Conc, concentration; TR, therapeutic range; AED, antiepileptic drugs; NR, not reported; CBZ, carbamazepine; PB, phenobarbital; PHT, phenytoin; OXC, oxcarbazepine; NS, not significant; VPA, valproate; TPM, topiramate; LTG, lamotrigine; PRM, perampanel; ETX, ethosuximide.

## Data Availability

The data presented in this study are available on request from the corresponding author (S.-Y.L.).

## Supplementary Materials

The following are available online at https://www.mdpi.com/article/10.3390/ph14080826/s1, Table S1: Co-medication drugs of 519 patients.
