# Supplementary material for: Levetiracetam Therapeutic Drug Monitoring in a Large Cohort of Korean Epileptic Patients
Source: Pharmaceuticals (Basel). 2021 Aug 23;14(8):826. doi: 10.3390/ph14080826 (PMC8401685; doi:10.3390/ph14080826)
Supplement: Supplementary file 1 [file pharmaceuticals-14-00826-s001.zip › pharmaceuticals-1316025-supplementary.pdf]

Supplementary Information

Table S1. Co-medication drugs of 519 patients.

Medication	*n* (%)
Valproic acid	156 (30.1)
Oxcarbazepine	151 (29.1)
Clobazam	136 (26.2)
Lamotrigine	89 (17.1)
Topiramate	78 (15.0)
Carbamazepine	74 (14.3)
Zonisamide	67 (12.9)
Pregabalin	46 (8.9)
Clonazepam	41 (7.9)
Lacosamide	40 (7.7)
Phenobarbital	34 (6.6)
Phenytoin	31 (6.0)
Perampanel	29 (5.6)
Lorazepam	11 (2.1)
Alprazolam	8 (1.5)
Diazepam	6 (1.2)
Midazolam	3 (0.6)
Rufinamide	3 (0.6)
Etizolam	2 (0.4)
Gabapentin	2 (0.4)
Vigabatrin	2 (0.4)
Ethosuximide	1 (0.2)
Others	
Antidepressant	24 (4.6)
Alzheimer/Parkinsonism medication	13 (2.5)
Antipsychotics	12 (2.3)
Miscellaneous	21 (4.0)
